# Carrageenan Based Bionanocomposites as Drug Delivery Tool with Special Emphasis on the Influence of Ferromagnetic Nanoparticles

**DOI:** 10.1155/2017/8158315

**Published:** 2017-02-20

**Authors:** Abida Kalsoom Khan, Ain Us Saba, Shamyla Nawazish, Fahad Akhtar, Rehana Rashid, Sadullah Mir, Bushra Nasir, Furqan Iqbal, Samina Afzal, Fahad Pervaiz, Ghulam Murtaza

**Affiliations:** ^1^Department of Chemistry, COMSATS Institute of Information Technology, Abbottabad 22060, Pakistan; ^2^Department of Environment Sciences, COMSATS Institute of Information Technology, Abbottabad 22060, Pakistan; ^3^Department of Biochemistry, Hazara University, Mansehra 21300, Pakistan; ^4^Faculty of Pharmacy, Bahauddin University, Multan, Pakistan; ^5^Faculty of Pharmacy and Alternative Medicines, Islamia University of Bahawalpur, Bahawalpur, Pakistan; ^6^Department of Pharmacy, COMSATS Institute of Information Technology, Abbottabad 22060, Pakistan; ^7^Institute of Automation, Chinese Academy of Sciences, Beijing 100190, China

## Abstract

Over the past few years, considerable attention has been focused on carrageenan based bionanocomposites due to their multifaceted properties like biodegradability, biocompatibility, and nontoxicity. Moreover, these composites can be tailored according to the desired purpose by using different nanofillers. The role of ferromagnetic nanoparticles in drug delivery is also discussed here in detail. Moreover, this article also presents a short review of recent research on the different types of the carrageenan based bionanocomposites and applications.

## 1. Introduction

In recent years, material chemists have fabricated advanced pharmaceuticals with improved biological, magnetic, and electrical characteristics. Nanoparticles have been studied as versatile drug delivery vehicles, particularly for targeted release of drug [1]. Accordingly, ferrous oxide nanoparticles (FENPs) possess excellent features such as superparamagnetism and low Curie temperature. Due to such characteristics, FONPs are extensively used as therapeutic tools, especially in cancer therapy [2]. Since FONPs are needed to be stabilized using suitable polymers [3], numerous studies on polysaccharide based magnetic nanocomposites have been reported [4].

Nanocomposites that contain naturally occurring polymers (biopolymer) in combination with an inorganic nanomoiety represent a separate class of materials called bionanocomposites (BNCs). The term “bionanocomposite” also called “nanobiocomposites” (NCs), “green composites,” or “biohybrids” was first used in 2004. Later on in 2011, there were about 66 articles in which this term was used [5, 6]. BNCs form an interesting interdisciplinary area that combines material sciences, biology, and nanotechnology [7]. Due to nanometer sized particles that are thoroughly distributed in the polymer matrix, these BNCs show improved mechanical, gas barrier, and optical properties [8, 9]. Moreover, BNCs exhibit significantly improved biodegradability and biocompatibility and also show functional properties that are being provided by biological or inorganic moieties [10]. BNCs are different from biocomposites that are partially made from biopolymers but they do not contain nanosize additives to inculcate the specific properties [7].

Biopolymers are the primary constituents for the synthesis of BNCs. These are classified as (i) polysaccharides, (ii) protein, (iii) DNA, and (iv) poly(hydroxyalkanoates); however polysaccharides and proteins are the most preferred groups.

### 1.1. Polysaccharides

Polysaccharides, also known as glycans, belong to carbohydrates. They can be obtained from various sources including seaweeds, plants, fungi, plants, insects, bacteria, crustacean, animals, and even humans [11]. They are subdivided into anionic and cationic polysaccharides. Carrageenan (CG) is a naturally occurring anionic sulphated polysaccharide extracted in 1837 from red seaweed of Rhodophyceae family, predominantly from* Chondrus crispus, Eucheuma, Gigartina stellata, Iridaea, Hypnea, Solieria, Agardhiella,* and* Sarconema *[12–14]. The word “carrageenan” is derived from Irish name* “carrageen”* meaning* “little rock”* [15]. It was first extracted by the people who lived in the vicinity of carrageen Ireland. Carrageenan is extracted by drying and washing seaweed in cool water. Seaweeds are then broken up and agitated in hot alkaline solution to extract CG. Once CG is in hot solution, it undergoes clarification and then is converted into powder [16].

Several methods have been used to remove CG from solution. First method is freeze thawing technique. In this method, solution is gelled with many salts and gels are frozen. Water is eliminated upon thawing and the resultant mass, that is, CG, and its salts are ground to the required particle size. Second technique is alcohol precipitation method. In this method, the concentrated solution of CG is placed in 2-propanol or other alcohols, which cause the precipitation of solution. Solvent is then evaporated and precipitated CG is ground to fine particles. Third method is KCl precipitation process in which after hot extraction the filtrate is evaporated and then extruded through spinnerets into cold solution of KCl. The resulting gel threads are then washed with KCl solution, pressed, dried, and milled to CG powder [17, 18].* Chondrus crispus, Gigartina stellata, Iridaea *spp.*, Eucheuma *spp., and* Kappaphycus *spp. are the prime raw materials used for CG extraction [16].

#### 1.1.1. Summary of Carrageenan Types, Structure, and Properties

**Figure 1:**
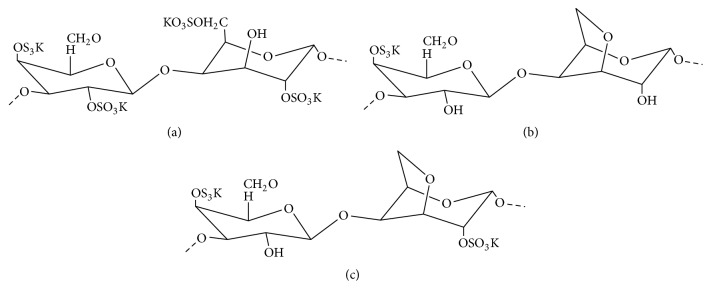
Structure of three types of carrageenan: (a) *λ*-CG, (b) *κ*-CG, and (c) ι-CG.

Carrageenan is found in several types in terms of chemical structure and properties, depending upon the species of seaweed used as a source. It is formed by alternate units of D-galactose and 3,6-anhydrogalactose that are joined by *α*-1,3 and *β*-1,4 glycosidic linkage. On the basis of type of bonding between galactose units and the position of attachment of sulphate groups to galactose unit, CG are categorized into *λ*- (lambda-) CG, *κ*- (kappa-) CG, ι- (iota-) CG, *υ*- (nu-) CG, *μ*- (mu-) CG, *θ*- (theta-) CG, and *ξ*- (Ksi-) CG, but on the basis of family, these are mainly of three types (Figure 1), ι-CG, *κ*-CG, and *λ*-CG [15, 16, 19, 20]. Among them, *λ*-CG has three sulphate groups per disaccharide repeating unit; *κ*-CG has one and ι-CG has two sulphate groups per disaccharide repeating unit. So, they have different linear charge density (*κ* < ι < *λ*) [20] and solubility (*κ* < ι < *λ*) [21]. They differ in the presence of the 3,6-anhydrobridges and number of sulphate groups.

**Figure 2:**
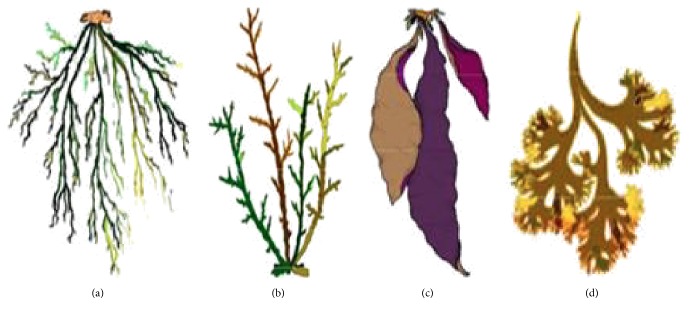
(a)* Eucheuma denticulatum-* (spinosum-) ι-CG. (b)* Kappaphycus alvarezii-* (cottonii-) *κ*-CG. (c)* Gigartina rodula*-*κ*-/*λ*-CG. (d)* Chondrus crispus*-*κ*-/*λ*-CG.

*κ*-CG is most abundant CG and* Chondrus crispus* constitutes 60% of CG. It is dissolved only in hot water and breaks down in acidic solution. Its structure shows that 3,6-anhydrobridges are present in *κ*- and ι-CG but absent in *λ*-CG [16, 21]. *κ*-CG is insoluble in milk and salt solution while forming strong gels in potassium salt. *λ*-CG is second most abundant form of CG and is major constituent of* Gigartina acicularis* and* Gigartina pistillata* (Figure 2) [16]. It does not contain 3,6 anhydro-D-galactopyranosyl; thus it does not form gels and is used as a thickener [21]. ι-CG is the least abundant and is found in* Eucheuma spinosum*. Gelling is the main property of *κ*- and ι-CG. *κ*-CG form brittle gels while ι-CG form soft and elastic gels. It is considered that the presence of anhydrobridges is responsible for gelation. Gels formed by the CG are thermally reversible [22]. In *κ*- and ι-CG the adjacent spiral chains containing sulphate group towards external part cross-link to form a network of three-dimensional double helix. Molecular weight of commercial CG ranges from 100 to 1000 kDa [21, 23].

Different properties of *κ*-, ι-, and *λ*-CG make these polymers a versatile tool to develop useful biomaterials. Carrageenan is an exceptionally versatile material having wide range of applications in food and other industries. Food applications of CG are due to its gelling, thickening, emulsifying, and stabilizing properties. In meat industry, it is used for the production of low fat potatoes and sausages [22, 24, 25]. It is also used in tooth paste, fire-fighting foams, air fresheners, shampoos, and the cosmetic creams [26].

Carrageenan has shown various pharmaceutical properties including anticancer, anticoagulant, antihyperlipidemic, and immunomodulatory activities [22, 27]. In vitro studies have revealed that CG may also have antiviral effects hindering the replication of hepatitis A virus [28]. In addition, other studies declared that CG effectively inhibits extensive range of sexually transmitted human papillomavirus [29]. Moreover, CG exhibits antioxidant activity also [30].

Because of its biocompatibility and consolidation behavior, CG is widely used by pharmaceutical scientists to improve drug formulation properties, especially to prolong drug release [31, 32]. It also helps to create pH/temperature sensitive drug delivery systems. When CG is used as sole matrix material in order to control drug release, the desired drug release profiles such as zero-order release and pH independent release cannot be obtained [33].

Particularly, CG based formulations are used for prolonged drug release, that is, for many hours or days [34, 35]. Some interesting features of CG like its adhesiveness and positive surface charge provide extra advantage in prolonging drug release in mucosal/epithelial tissues [36].

The interaction of CG with other polymers is used to attain ideal drug release profile. It has been studied that *κ*-CG showed fast release because of fast degeneration [37] or quick swelling [31, 32]. The blending of different types of CG with each other also appears to be a good strategy to prolong drug release, although SEM studies showed that some CG are not miscible in blends [38]. The tablets formed by equal amount of ι- and *λ*-CG showed zero-order release profile of tripelennamine-HCl. It can be explained as that *λ*-CG can encave the complexes of drugs and hydrated ι-CG in viscous shell of *λ*-CG [39]. This represents a smart “collaboration” between different types of CG in rational design of drug delivery system. The blending of CG with nanoparticles also enables prolonging drug delivery but different types of nanoparticles exhibit different effects. For example, the incorporation of gold nanofillers slows down the release rate of model drug (methylene blue: MB) [40]. In recent times, the applications of CG based formulations have extended to buccal [41], ophthalmic [42], and vaginal [43] drug delivery systems, as well as wide-ranging areas of wound healing [44] and tissue engineering [45].

In addition, *κ*-CG was also explored as the matrix of controlled release tablets. Using microcrystalline cellulose as the filler and theophylline monohydrate as model drug (20%), the effect of *κ*-CG content on drug release was studied. It was observed that 20% (v/v) *κ*-CG resulted in fast drug release. Slower release was observed at 30% (v/v) *κ*-CG content while at 70% (v/v) *κ*-CG, drug release followed zero-order kinetics [31, 32]. Other studies demonstrated that *κ*-CG can interact with chitosan in the presence of cross-linker tripolyphosphate (TPP) to form stable nanoparticles. The size of these nanoparticles and their positive surface charge is ideal for penetrating epithelial surface [46].

Water absorption capacity is another very simple and useful feature of CG polymers, which increases drug dissolution and thus increases the oral bioavailability of poorly water-soluble drugs [47]. Thus it can be used as a practical alternative to microcrystalline cellulose (MCC) pellet or gelatin capsule. In comparison to MCC pellets, the pellets fabricated with *κ*-CG enable water-insoluble drugs to collapse and then dissolve as quick as 20 min [47–49]. Taken together, the unique properties of all three CG varieties are now benefiting both oral and parenteral drug release in various ways.

#### 1.1.2. Role of Iron Oxide Nanoparticles in Magnetically Guided and Magnetically Responsive Drug Delivery

Magnetic nanoparticles (MNPs) are one of the most commonly used nanoscale materials [50, 51]. These are the core/shell type nanoparticle structures that consist of a magnetic core, which is encapsulated in an organic or polymeric coating. MNPs have hydrophobic surface with large surface-to-volume ratio, so they agglomerate in the absence of surface coating [52]. MNPs have unique magnetic phenomenon that is extremely different from their bulk counterparts. Since their properties can be employed in variety of applications, extending from storage media for magnetic memory devices to probes and vectors in biomedical sciences [53].

The large surface-to-volume ratio of magnetic nanoparticles provides abundant chemical active sites for biomolecule conjugation [54, 55]. Their magnetic properties allow MNPs to be used in many applications related to drug and gene delivery, therapeutics and diagnostics. Besides their size, surface properties of MNPs are also very essential for their applications. Coating of MNPs with external layer of different materials provides an interesting approach for transforming their surface properties. Variety of coating materials are used to modify the surface chemistry of MNPs like organic polymers (dextran, chitosan, polyethylene glycol (PEG), polysorbate, and polyaniline), organic surfactants (sodium oleate and dodecylamine), inorganic materials (gold, silica, and carbon), and bioactive molecules (liposomes, peptides, and ligands/receptors). Coated nanoparticles have advantages over simple nanoparticles like they have less cytotoxicity, show increased dispersibility and biocompatibility, have better conjugation with other bioactive molecules, and have improved thermal and chemical stability [56, 57]. For in vivo studies, MNPs should have high magnetic saturation. For instance, nanoparticles with high magnetization are required as contrast agents for MRI. Mainly iron oxide nanoparticles are used for many applications. Iron oxide exists as magnetite (Fe_3_O_4_) or maghemite (*γ*-Fe_2_O_3_).

**Figure 3:**
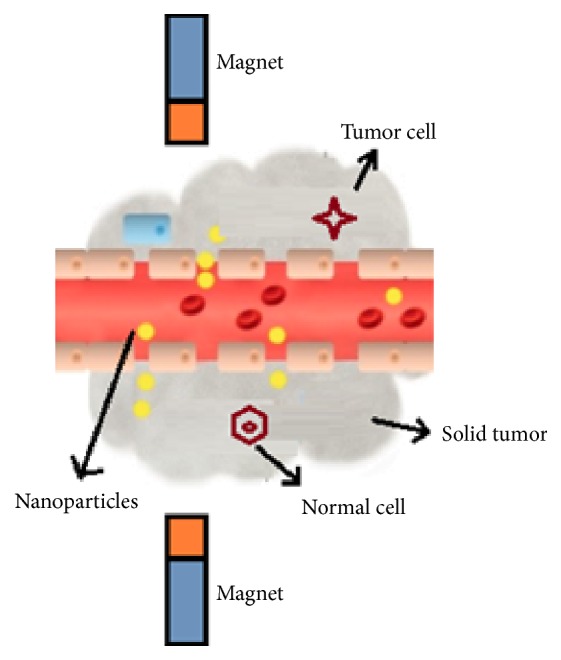
Magnetically guided drug targeting.

*(1) Magnetically Guided Drug Targeting. *Freeman et al. proposed the idea of magnetically guided drug targeting (MGDT). According to him, MNPs can be transported through vascular system to a particular point in the body with help of magnetic field (Figure 3). The method of MGDT involves the immobilization of drug in to MNPs under the effect of magnetic field gradient, followed by the injection of drug or drug carrier complex into the living body either via intravenous or intra-arterial injection, and finally, the use of high-gradient external magnetic fields generated by rare-earth permanent magnets to guide the complex and concentrate it at the chosen target locations. Once the complex is concentrated at target in vivo, therapeutic agent is released from the magnetic carrier, either by enzymatic activity or via changes in physiological conditions such as pH, osmolality, or temperature. This results in increased uptake of drug by tumor cells at target sites [58] and a restricted systemic drug concentration [59].

The improvement in magnetically guided nanoparticles allows them to accumulate at particular pathologies such as inflammatory, tumors, and infectious sites. Those pathologies are characterized by structural abnormalities in the vasculature. This phenomenon is known as the enhanced permeability and retention (EPR) effect [60]. The method of MGDT depends not only on physical properties, concentrations, and the amount of particles applied but also on the type of binding of drugs. In addition, strength, geometry, and time interval of external magnetic field also affects MGDT. It also depends on the route of MNPs injection and the vascular supply to the targeted tissues. 

*(2) Magnetically Induced Drug Release.* The idea of magnetically induced drug release (MIDR) was introduced by Kost et al. [61], who first used the idea of external magnetic field in order to achieve pulsatile release from polymer composites. He observed the insulin release from a magnetic composite of an ethylene-vinyl acetate copolymer by using low-frequency applied magnetic field (AMF). Even the inherent thermal energy from MNPs can also be used as an external and remote control trigger to control drug release. This energy opens up the gate for organic or inorganic carrier that contains drug for therapy. The first studies applied microwave radiation to liposomes with enwrapped ferromagnetic microparticles to stimulate drug release [62]. The applied magnetic field can suppress drug-drug carrier interactions and speed up diffusion [63]. In the presence of AMF, drug release rate is considerably enhanced, because the mechanical deformation of pulsatile system generates compressive and tensile stresses. Furthermore, AMF-triggered drug delivery systems use the collapse or volume transition of drug carriers to induce drug release [64].

Liposomes provide a way of scattering and concentrating nanoparticles and drug through encapsulation or binding. The simple way to deliver drug from liposomes is diffusion through lipid bilayer. Diffusion is increased when the bilayer is phase separated or disrupted. Thus, the permeability of the bilayer affects the diffusion of drug, and the permeability of liposomes is highly increased around the membrane melting temperature (*T*m), which rely on the lipid composition [65]. The cargo can be released if the liposome membrane is heated above* T*m. Mechanistically, the heating intensity under high frequency magnetic field is controlled by the mode of magnetic energy decadence for single domain particles owing to two reasons: (i) external Brownian shifts and (ii) internal Neel fluctuations of the particle magnetic moment [66]. In order to use liposomes as thermoresponsive drug delivery vehicle,* T*m is designed typically close to body temperature, to release the cargo at few degrees higher than the temperature of pathological tissue, such as cancer. Under such conditions, liposome leakage is produced during circulation to trigger cargo release under the effect of high frequency AMF. Magnetoliposomes were the first multifunctional hybrid liposome/nanoparticle assembly that received significant attention since 1988 [67].

**Figure 4:**
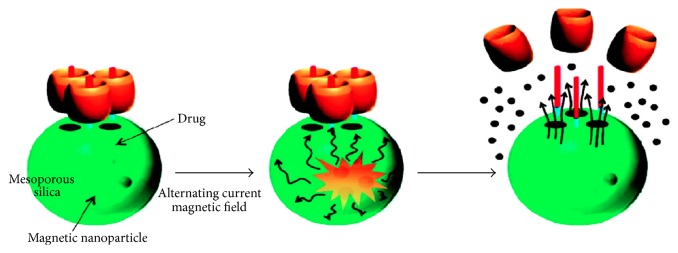
Magnetically triggered drug release system.

Another approach for synthesis of polymeric MNPs includes the precipitation of MNPs within a porous polymer microparticles or nanoparticle scaffold [68]. One advantage of this technique is that particles with a relatively narrow size distribution can be produced, and well-defined spherical morphology can also be achieved (Figure 4). Specifically, 15 nm FONPs are inserted into the porous drug carrier with molecular valves [69]. A molecular valve, consisting of thread and capping molecule, blocks the silica pores to keep the drug inside. On applying an external AMF, the generation of heat and subsequent pressure buildup (~90 bar) inside the porous nanoparticles causes the fast removal of molecular valves leading to release of the cargo. These materials are valuable for in vivo drug delivery such as release of doxorubicin (DOX) in breast cancer cells [69]. Hence, the development of MNPs and their use as drug carrier have attracted enormous attention. Major advantages of magnetofection include its simplicity, a modest cost, enhanced localization, efficient delivery, and drop in both incubation time and vector doses. The promising in vivo results have been reported for preclinical trials of gene transfection in cats for the treatment of feline fibrosarcomas. Meantime, magnetically sensitive MNPs establish a platform that shows the highest diversity in drug delivery field. Apart from the use of thermosensitive liposomes and gels that are sensitive to heat produced by the action of interchanging radiation on MNPs, other systems have produced encouraging results.

## 2. Applications of Carrageenan Nanocomposites

**Figure 5:**
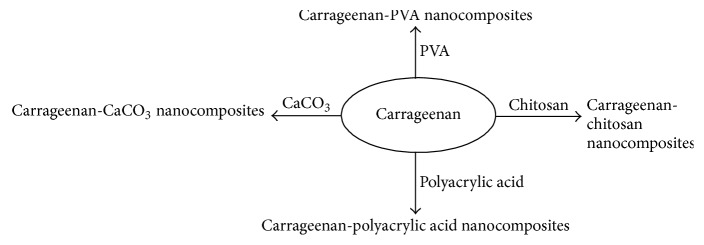
Carrageenan nanocomposites reported in literature.

From the last few decades, scientists are putting their efforts to explore the applications of CG in biomedical field. Blending of nanoparticles (NPs) with CG enhances its properties and extends its use in pharmaceutical and biomedical applications. That is why use of CG based biomaterials is increasing (Table 1). So, there is a need to carefully analyze the properties of CG in order to use this type of polymer in broad range.  Figure 5 presents various carrageenan nanocomposites reported in literature.

### 2.1. Nanocomposites Containing Carrageenan Only

Controlled drug delivery has attracted so much attention of pharmaceutical formulators. It is one of the greatest challenging therapeutic strategies for the treatment of chronic diseases. In this regard, biopolymers are proved very advantageous. In past few decades, CG based bionanocomposites have increasingly been used for pharmaceutical purposes. Carrageenan due to its gelling property improves the drug formulation and sustained release.

The interaction between filler components of nanocomposites at the nanometer scale enables them to act as molecular bridges in the polymer matrix: in fact it is the main reason for enhanced mechanical properties of the nanocomposite as compared to conventional microcomposites. Bionanocomposites add a new dimension to these enhanced properties due to the biocompatible and/or biodegradable nature of the material.

The incorporation of MNPs in polysaccharides is also being explored. Unique properties of ferrous oxide nanoparticles (FONPs) make nanocomposites a promising material for use in biomedical applications and form hydrogel nanocomposites that are responsive to external magnetic field. An important advantage of these magnetic NPs is the magnetically driven transport of drug which allows site specific drug delivery [84]. Moreover by changing the external magnetic field, a remotely controlled release of encapsulated therapeutic agents can also be achieved [85, 86]. Hence, such type of magnetic hydrogel nanocomposites is very efficient for the development of site specific or time controlled drug delivery systems [87, 88]. In addition, MNPs also confer new functionalities to the resulting nanocomposites that are valuable for other biomedical applications like medical imaging [89, 90].

Impact of magnetic ferrous oxide on swelling behavior and release properties of MB has been studied by Daniel-Da-Silva and the companions. In this regard, *κ*-CG based hydrogel nanocomposites have been synthesized. *κ*-CG was used as matrix and magnetic FONPs have been chosen as nanofiller and MB was used as model drug. It has been noted that addition of MNPs and increase in its concentration causes increased swelling ratio and forms stronger gels, which affect oppositely the release rate of MB. The release kinetics of MB depends upon the FONPs load. So, incorporation of nanoparticles to polyelectrolyte hydrogel is a valuable way to tailor the release rate of encapsulated drugs [70].

Raman et al. explained the synergistic effect of ι-CG and MNPs in drug delivery. In this study, *γ*-FONPs were electrostatically entrapped in ι-CG to develop a nanocomposite. The prepared nanocomposites were characterized by various analytical techniques and then in vitro analyses were made. These in vitro analyses reveal that the synthesized nanocomposites are potential candidate for cancer therapy due to apoptosis. This was confirmed by Hoechst 33342 and 7-AAD staining studies under fluorescent microscopy. MTT assay confirmed the biocompatibility of nanocomposites against normal cells. Cell apoptosis was induced by following the ROS-mediated mitochondrial pathway combined with downregulation of expression levels of mRNA of XIAP and PARP-1 and upregulation of caspase-3, Bcl-2, and Bcl-xL [78].

Moreover in 2015, other scientists like Shanmuga and his coworkers also prepared *κ*-CG loaded MNPs for the same purpose [91]. Other researchers also prepared *κ*-CG/gold (Au) nanocomposites and observed that spherical and rod shaped Au-NPs cause controlled diffusion of MB [40]. The incorporation of Au-NPs seems to slow down the rate of diffusion of MB molecules. This is probably due to the impact of the Au-NPs on the tortuosity of CG matrix. Tortuosity is a parameter that accounts for contribution of average pore size, distribution of pore size, and the pore interconnectivity of hydrogel which is inversely related to diffusion coefficient [92]. DSC confirmed that the incorporation of Au-NPs originates a more heterogeneous polymer network than in the original hydrogel. This causes changes in the tortuosity of CG matrix. An increases in tortuosity would cause smaller MB diffusion rate that could lead to a diffusion controlled mechanism [93, 94]. The anisotropy of gold nanofillers was found to affect the aggregation of CG helices, which render microstructures of hydrogel less homogeneous. For the equivalent content of Au-NPs, the release kinetics of MB depends on the nature of Au-NPs that are used as the dispersed phase. But MB release from CG hydrogels occurred through anomalous transport. It tended to be controlled by polymer relaxation and erosion, if spherical Au-NPs were incorporated. Considering that the addition of Au-NPs originates stronger hydrogels, it would be expected that MB release followed a polymer relaxation mechanism irrespective of the shape of the Au-NPs [40].

Metal nanoparticles having high surface area have been extensively used because of their unique physiochemical properties including electronic properties, magnetic characteristics, antimicrobial activity, catalytic activity, and biomedical applications. These are also known to have bactericidal effects [95, 96].

Hence, Hezaveh and Muhamad investigated that addition of MgO nanoparticles in *κ*-CG hydrogel nanocomposites positively affects the release of MB in gastrointestinal tract (GIT). This concept is based on the fact that there are various substrates that could be known by anaerobic bacteria and are destroyed by enzymes in the colon [97]. So, it is valuable to fabricate polysaccharide based new materials that are stable in gastric environment and the colon targeted delivery. These NPs keep the loaded drug in their interphase with nanocomposite structure. When the nanocomposites are in contact with medium solution, these nanosized drug reservoirs release the entrapped MB; hence it acts as drug releasing channels for MB within the hydrogel network. That is why drug release in nanocomposite hydrogels is higher than that of blank gels. It has been observed that less MB was released in stomach. This is due to the fact that, at low pH, a compact network blocks the interphase region between the nanoparticles and the nanocomposite network [73, 74].

He also used silver and magnetic nanofillers in *κ*-CG nanocomposite hydrogels for the controlled release of MB in GIT. The nanocomposites containing silver (Ag) NPs showed profound MB release in acidic medium than intestinal medium while magnetite nanocomposite hydrogel exhibited better performance. High cumulative drug release of Ag-NPs loaded hydrogel can be accredited to the presence of Ag-NPs colloids with high surface charges in the network [73]. Moreover, increased concentration of NPs causes an increase in MB release and fine distribution of these NPs leads to more control over MB and causes smoother release. To increase the performance of nanocomposite hydrogels, the effect of natural cross-linking agent genipin was also investigated. Addition of cross-linker causes decreased MB release as it affects the porosity and structure of hydrogel network. Generally, cross-linking results in denser and more rigid hydrogel; the pore size decreases leading to the reduction in degree of swelling in aqueous medium. Cross-linking had a positive impact on drug release property and it has been seen that more targeted release was achieved due to genipin cross-linking [73, 74]. Studies revealed that addition of genipin cross-linker also enhances the physical properties of polymer network which improves the controlled release property of nanocomposite hydrogel [97].

One of the most challenging subjects of research in food industry is to prepare environment friendly packaging material with additional antimicrobial properties for food safety and to increase its shelf life. Bionanocomposites with antimicrobial activity are most promising for this purpose [98–100]. The use of natural biopolymers for making biodegradable edible films has increased. Carrageenan has frequently been used for biodegradable packaging film preparation. To improve their properties, variety of nanofillers is used. Inorganic based biodegradable hydrogels are more capable for the inactivation of bacteria due to which they are mostly used in biomedical and biotechnological field [101].

Jayaramudu and his coworkers synthesized Ag-nanocomposite hydrogel with ι-carrageenan through green process. Silver nanoparticles were prepared by reduction of silver nitrate (AgNO_3_) in* Azadirachta indica* leaf extract in hydrogel network. These nanocomposite hydrogels were characterized by UV-Vis spectroscopy, X-ray diffraction spectroscopy, and other analytical techniques. The Ag-nanocomposite showed high swelling ratio because addition of Ag-ions led to the formation of NPs within the hydrogel system that expanded the gel networks and increased the water molecules uptake capacity. In addition, the antibacterial activity of biodegradable Ag-nanocomposite hydrogel was investigated against* Bacillus* and* Escherichia coli*. A strong zone of inhibition was seen against these species [79].

Another antimicrobial bionanocomposite film was synthesized by Rhim and Wang by using *κ*-CG, Ag-NPs, and organically modified nanoclay as a nanofiller. Addition of nanoclay increases the mechanical strength of these films. The films containing only Ag-NPs show strong antimicrobial activity against Gram negative bacteria [102]. The antibacterial action of Ag-NPs has not been established yet, but various mechanistic actions have been reported. First is the cell membrane disruption because of contact of Ag-NPs with sulfur and phosphorous containing compounds of DNA and proteins, which prevents DNA replication leading to cell death [103]. Second is the binding of the positive Ag-NPs with negatively charged bacterial cell membranes which disrupts the cell wall and surface proteins causing cell death [80]. Third is the penetration of Ag-NPs into the bacteria, which deactivates enzymes and yields H_2_O_2_ leading to cell death [104], while clay containing nanocomposites presented strong antibacterial activity against Gram-positive bacteria. The antimicrobial activity of clay is due to the presence of alkyl quaternary ammonium salt group in clay mineral. The synergistic effect of both nanofillers displayed potential antimicrobial activity against both Gram-positive and Gram negative bacterial strands [102].

Shankar et al. reported that chitin nanofibrils (CNF) reinforced *κ*-CG nanocomposites also show strong antimicrobial activity against Gram-positive food borne pathogens* (Listeria monocytogenes)*. These films showed distinct antimicrobial activity against* Listeria monocytogenes* and seemed dependent on CNF concentration. The antibacterial activity of the CNF is probably due to the fact that it makes bacteria flocculate and inhibits growth through lack of nutrients and oxygen [81].

Later on, montmorillonite induced *κ*-CG films were developed by Shojaee-Aliabadi et al. using* Zataria multiflora* Boiss. essential oil, which acted as hydrophobic agent. The antimicrobial activity of prepared films was tested against five pathogens, that is,* Staphylococcus aureus (S. aureus), Bacillus cereus, Escherichia coli, Salmonella typhimurium, *and* Pseudomonas aeruginosa* through overlay and vapor phase process. Results showed that *κ*-CG/MMT (montmorillonite)/ZEO (*Zataria multiflora* essential oil) films showed strong antimicrobial activity against all pathogens. *κ*-CG/nanoclay films did not show any inhibition against these tested bacteria [82]. Previous studies have also reported good inhibitory effect of ZEO against* Salmonella typhimurium, Escherichia coli, Bacillus cereus, or Staphylococcus aureus* [45, 46], but no information has been obtained about the activity of ZEO against* Pseudomonas aeruginosa*. The synergistic effect of both ZEO and nanoclay on antimicrobial, mechanical, and water vapor permeability properties was also noted [82].

Hosseinzadeh synthesized hydrogel nanocomposites using CG and multiwalled carbon nanotube (CNTs). These were prepared by in situ graft polymerization of acrylic acid in the presence of methylene bisacrylamide as cross-linker. The adsorption ability of hydrogel nanocomposite was inspected to remove crystal violet (CV) as a cationic dye. It was seen that the adsorption capacity of nanocomposites varies with multiwalled carbon nanotube (MCNTs) concentration and agitation time as well as the pH of solution. The maximum adsorption capacity of CG/MCNTs hybrid hydrogel nanocomposite was found to be 118 mg/g [83].

### 2.2. Nanocomposites Containing Carrageenan and PVA

Moreover Mahdavinia and his coworker also investigated the influence of MNPs and *κ*-CG on swelling capacity and drug release property of interpenetrating polymer network (IPN) nanocomposite hydrogel composed of CG, polyvinyl alcohol, and FONPs. The produced polymer hydrogels loaded with diclofenac sodium (DS) were cross-linked by freezing thawing technique. The release study of DS revealed that drug release not only is pH dependent but also depends on magnetic field [72]. It was seen that amount of released drug was increased by increasing the strength of applied magnetic field. It has been suggested that, at low magnetic field, the magnetic moment of MNPs aligned together and led to the accumulation of FONPs. This aggregation of MNPs decreases the pore size of magnetic hydrogel which in turn decreases the diffusion of drug [105]. While at high applied magnetic field, larger diffusion of drug occurred. At different pH, drug release was different due to difference in its solubility. Minimum drug release was observed at phosphate buffered saline (PBS) solution having pH 1.2 because of low solubility of DS in acidic medium while maximum drug release was seen at PBS media with pH = 7.4.

It has also been proved that *κ*-CG based IPN nanocomposite hydrogels have the capability to inactivate the Gram-positive* S. aureus *bacteria and the size of zone of inhibition was affected by the amount of drug in hydrogels [71].

### 2.3. Nanocomposites Containing Carrageenan and Chitosan

Furthermore, they had also reported dual natured magnetic and pH responsive hydrogel beads for controlled drug release. These hydrogel beads were derived from *κ*-CG/carboxymethyl chitosan nanocomposites. FONPs were synthesized inside the polymers via in situ process. The prepared hydrogel beads showed maximum swelling at pH 7.4, while the addition of MNPs decreases it. The drug loading and release of DS showed that drug release is effected by pH and is increased by applying external magnetic field. At pH 1.2, minimum drug release was seen. This is because of low solubility of DS at this pH which is due to the existence of weak carboxylic acid group on drug with pKa of 4, while a large amount of drug release occurred at pH 7.4 because the swelling of hydrogel beads increased significantly due to the neutralization of carboxylic acid groups on beads producing anionic carboxylate. The prepared beads showed magnetic properties. By applying external magnetic field, the amount of drug release varied. On applying external magnetic field, MNPs aligned with the AMF due to which the drug release increases. It occurs actually due to the continuous motion of MNPs. The fluctuation of AMF acts as stimulus to agitate the MNPs. The motion of MNPs increases which leads to relaxation of polymeric backbone causing an increased amount of released drug [106, 107]. Further increasing the strength of AMF, the amount of dug release also increases [72].

Long and his coworkers evaluated the potential of magnetic chitosan/CG nanocomposites for controlled release of macromolecules like protein. Magnetic CG nanocomposites were prepared at ambient conditions which were then incorporated into chitosan solution to gain CS/CRG nanocomposites. At the end, bovine serum albumin (BSA) was loaded. Due to the viscosity effect of CG, magnetic CG nanocomposites showed weak response to the AMF. That is why chitosan is used to prohibit cross-linking between the nearby chains of CG and to neutralize the charge of sulphate groups in particular surface. The loading capacity and release profile of the prepared nanocomposites demonstrated sustained release of protein in intestinal medium. About 85% of protein was released in 30 minutes. Moreover, at high pH, a large amount of BSA was released because at high pH, that is, 7, chitosan becomes insoluble due to which weak electrostatic interaction exists between chitosan and BSA. The zeta-potential of chitosan decreased with increase in pH. Hence, the BSA was released at pH 7.4, but not at pH 1.2 or 6.8 [77].

### 2.4. Nanocomposites Containing Carrageenan and Polyacrylic Acid

Later on in 2013, *Κ*-CG-g-poly(acrylic acid)/SPION (superparamagnetic iron oxide nanoparticles) nanocomposite were prepared by Bardajee et al. for the controlled release of deferasirox. These nanocomposites were prepared by simultaneous development of SPION and the cross-linking of poly(acrylic acid) grafted onto the *κ*-CG (*κ*C-g-PAA). The swelling property of obtained hydrogels was measured at different conditions. In vitro studies of *κ*-CG-PAA/SPION hydrogel studied were made at different temperature, pH, and magnetic field. The rate of drug release was increased with increase in pH and temperature. While in the absence of external magnetic field (EMF) at pH 7, higher drug amount was released as compared to that of in the presence of EMF. This is due to the formation of close configuration of hydrogel because of attractive forces between SPIONs and a quick decrease in the porosity volume of hydrogel. Thus, the drugs are strongly kept in the network of hydrogel, due to which diffusion of drug from the hydrogel is reduced. The biocompatibility studies also reveal that the prepared hydrogels are nontoxic and hence useful for biomedical applications [75].

### 2.5. Nanocomposites Containing Carrageenan and Calcium Carbonate

As biopolymers are proved very advantageous for the controlled delivery of drugs, Bosio and his coworkers developed calcium carbonate based hybrid nanoporous NPs. His objective was to develop hybrid NPs (hNPs) to deliver DOX to target cancer cells. The biopolymers used were derivatized with folic acid, since targeting of cancer cells is feasible by coupling *λ*-CG to folic acid in the microparticles (FA-*λ*-Car-hNPs). The specific surface area is also increased to ninefold. The anticancer activity of FA-*λ*-Car-hNPs was tested on human osteosarcoma MG-63 cells which indicated higher cytotoxicity and thus used as potential candidate for cancer therapy [76].

## 3. Conclusion

All the major three types of carrageenan and their nanocomposites provide diverse applications in pharmaceutical industry encompassing the delivery of small chemical drugs, proteins, and cells, as well as tissue regeneration using therapeutic biomacromolecule. The addition of nanoparticles in carrageenan composites enhances its pharmaceutical properties. The increasing information of structural analysis and chemical modifications of carrageenan allows the better utilization of carrageenan as probe to investigate various dangerous diseases, such as cancer and AIDS.

## Figures and Tables

**Table 1:** Important findings of previous studies involving carrageenan bionanocomposites.

Sr. number	Combination polymer	Objective	Important findings	References
(1)	*κ*-Carrageenan	To examine the effect of FONPs on swelling, kinetics, and drug release mechanism.	Addition of MNPs causes high swelling ratio and forms stronger gels. The release rate of model drug can be tailored with the concentration of MNPs.	[70]
(2)	Poly vinyl alcohol	To control the release of drug (diclofenac sodium) via MNPs.	Drug release depends on pH and magnetic field.	[71]
(3)	Carboxymethyl chitosan	To modify drug release pattern.	Increase in drug release by applying external magnetic field as well as elevating of pH	[72]
(4)	Carrageenan	To enhance the performance of carrageenan hydrogels as drug delivery carrier in gastrointestinal conditions.	Less release of methylene blue in stomach.	[40]
(5)	*κ*-Carrageenan	To develop new nanocomposite hydrogels via in situ approach to find a suitable drug carrier for GIT release.	MB release increased with increased concentration of NPs.	[73, 74]
(6)	Poly(acrylic acid)	To produce novel biocompatible triple-response hydrogels based on *k*-CG.	Higher drug release in the absence of EMF at pH 7.	[75]
(7)	Calcium carbonate (CaCO_3_)	To fabricate and characterize hybrid microparticles (hNPs) to deliver doxorubicin against cancer cells.	Coupling of *λ*-CG to folic acid increased the targeting of cancer cells.	[76]
(8)	Chitosan	To evaluate the release potential of natural polymer coated MNPs for controlled release of macromolecules.	Greater release of BSA at high pH.	[77]
(9)	None	To explore the synergistic effect of ι-CG and MNPs in drug delivery and cancer therapy.	Prepared nanocomposites proved to be potential candidate for cancer therapy due to apoptosis.	[78]
(10)	None	To explore the antibacterial applications of inorganic biodegradable hydrogels.	A strong zone of inhibition against *Bacillus* and *Escherichia coli*.	[79]
(11)	None	To formulate environment-friendly nanocomposite films comprising of carrageenan, AgNPs, and clay mineral to investigate their combined effect on antimicrobial activity and physicochemical film properties.	The combined use of both nanofillers (AgNPs and clay) showed potential antimicrobial activity against Gram-positive and Gram-negative bacteria.	[80]
(12)	None	To synthesize CG/CNF nanocomposite films and to study the effects of CNF concentrations on various properties of CG/CNF nanocomposite films.	Strong antimicrobial activity of the prepared films against Gram-positive food borne pathogens *(Listeria monocytogenes)*.	[81]
(13)	None	To enhance the physical barrier and mechanical properties of CG based films by the addition of nanoclay as well as to check the antimicrobial effect of ZEO added in these films.	Strong microbial activity against *S. aureus, B. cereus, E. coli, S. typhimurium, *and *P. aeruginosa.*	[82]
(14)	Carbon nanotubes (CNTs)	To prepare CG-based hydrogels impregnated with CNTs and evaluate their swelling behavior and adsorption performance of crystal violet (CV) as model dye.	Lower adsorption of CV at acidic pHs and high adsorption at high pH. Moreover adsorption of CV also increases with increase in concentration of MCNT.	[83]
